# Replication characteristics of porcine reproductive and respiratory syndrome virus (PRRSV) European subtype 1 (Lelystad) and subtype 3 (Lena) strains in nasal mucosa and cells of the monocytic lineage: indications for the use of new receptors of PRRSV (Lena)

**DOI:** 10.1186/1297-9716-44-73

**Published:** 2013-09-04

**Authors:** Ilias S Frydas, Mieke Verbeeck, Jun Cao, Hans J Nauwynck

**Affiliations:** 1 Laboratory of Virology, Department of Virology, Immunology and Parasitology, Faculty of Veterinary Medicine, Ghent University, Salisburylaan 133, Merelbeke B-9820, Belgium

## Abstract

Recently, it has been demonstrated that subtype 3 strains of European type porcine reproductive and respiratory syndrome virus (PRRSV) are more virulent/pathogenic than subtype 1 strains. This points to differences in the pathogenesis. In the present study, a new polarized nasal mucosa explant system was used to study the invasion of the low virulent subtype 1 PRRSV strain Lelystad (LV) and the highly virulent subtype 3 PRRSV strain Lena at the portal of entry. Different cell types of the monocytic lineage (alveolar macrophages (PAM), cultured blood monocytes and monocyte-derived dendritic cells (moDC)) were enclosed to examine replication kinetics of both strains in their putative target cells. At 0, 12, 24, 48 and 72 hours post inoculation (hpi), virus production was analyzed and the infected cells were quantified and identified. Lena replicated much more efficiently than LV in the nasal mucosa explants and to a lesser extent in PAM. Differences in replication were not found in monocytes and moDC. Confocal microscopy demonstrated that for LV, almost all viral antigen positive cells were CD163^+^Sialoadhesin (Sn)^+^, which were mainly located in the lamina propria of the respiratory mucosa. In Lena-infected nasal mucosa, CD163^+^Sn^+^, CD163^+^Sn^-^ and to a lesser extent CD163^-^Sn^-^ monocytic subtypes were involved in infection. CD163^+^Sn^-^ cells were mostly located within or in the proximity of the epithelium. Our results show that, whereas LV replicates in a restricted subpopulation of CD163^+^Sn^+^ monocytic cells in the upper respiratory tract, Lena hijacks a broader range of subpopulations to spread within the mucosa. Replication in CD163^+^Sn^-^ cells suggests that an alternative entry receptor may contribute to the wider tropism of Lena.

## Introduction

Porcine reproductive and respiratory syndrome (PRRS) is the most devastating disease in swine-producing countries all over the world with huge annual economic losses [1]. PRRSV causes reproductive failure in late gestation sows and is associated with respiratory disorders in growing pigs. It is a positive single stranded enveloped RNA virus classified together with lactate dehydrogenase virus, simian hemorrhagic fever virus and equine arteritis virus in the family of the *Arteriviridae* within the order *Nidovirales*[2]. The syndrome was first detected in the United States in the late eighties and the virus was isolated in Europe, America and Asia in the early nineties [3,4]. Genetic analysis of PRRSV isolates from all over the world showed the existence of two major types with clear genetic and antigenic differences: European type 1 (prototype Lelystad - LV) and American type 2 (prototype VR2332) [5,6]. During the last decade new highly virulent strains emerged in Eastern Europe, China and Vietnam, showing more severe clinical signs [7,8]. Previous in vivo studies showed for LV a narrow cell tropism limited to cells of the monocytic lineage that are present in lungs, lymphoid tissues, placenta and other organs [9,10]. In vitro studies showed that freshly isolated blood monocytes, red bone marrow cells and peritoneal macrophages were resistant to infection with LV, whereas cultured blood monocytes, monocyte-derived dendritic cells (moDC), red bone marrow-derived dendritic cells (BmDC) and primary alveolar macrophages (PAM) were susceptible to type I and II PRRSV [11-15]. The host cell receptors of PRRSV were identified in PAM as sialoadhesin (Sn) and CD163, responsible for virus attachment, internalization and disassembly, both of which are exclusively expressed by different types of monocytic cells [16-19].

Airway mucosal surface is a common entry site for many viruses. In evolution, these pathogens have developed several different mechanisms to penetrate through the epithelial cell layer, degrade the basement membrane and spread in the underlying connective tissue [20]. The epithelial cell layer forms a strong barrier against pathogens primarily due to cell-cell junctions and desmosomes [21]. Due to the complex nature of the mucosal surfaces, tissue cultures are preferred to study pathogen replication and invasion. Tissue cultures of mucosa of different species have already provided new insights in the pathogenesis of early stages of infection with alphaherpesviruses (PrV, EHV, BoHV, HSV) and retroviruses (HIV) [22-26].

PRRSV spreads by direct contact through saliva, nasal and mammary secretions, urine, feces and semen and transplacental transmission in late gestation [10]. In addition, an aerogenic spread over a distance of 4 km has also been reported. This aerosol transmission of the virus is mainly influenced by the pathogenicity of the isolate [27]. Despite the increasing knowledge of the pathogenesis of PRRSV, the pathways that the virus follows to invade at mucosal surfaces at the early stage of infection remain unknown.

In the present study, the mechanism to penetrate the nasal mucosa was examined for PRRSV. To this end, polarized nasal mucosa explants, which were embedded in agarose in order to represent the natural condition of the respiratory epithelial barrier, were used. Replication characteristics and cell tropism of a low virulent European subtype 1 strain (LV) and a new highly virulent European subtype 3 strain (Lena) were compared. Lena, was isolated by Karniychuk et al. [8], and alignment with LV showed 84% identity and 89% similarity at the amino acid level, with the greatest variation in Orf1a (Nsp2) where a deletion of 29 amino acids was found [28]. In addition, viral replication of both strains was compared in different types of monocytic cells.

## Materials and methods

### Animals and collection of tissue and cells

**Figure 1:**
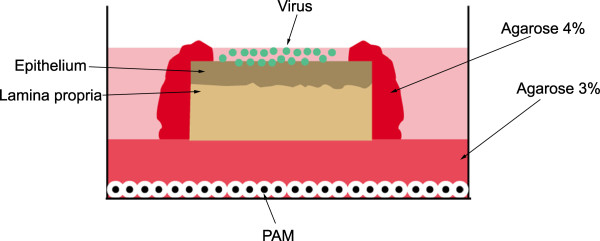
**An illustration of the polarized tissue explant model for studying PRRSV replication kinetics.** Nasal mucosa explants were placed over a 3% agarose layer with the mucosal side facing up in a 6-well plate. The lateral edges of the explants were sealed with 4% agarose and cell-free PRRSV particles were added to the apical surfaces. PRRSV susceptible cells (PAM) were seeded on the bottom of the plate to examine virus particle penetration through the agarose layer. PAM: Porcine alveolar macrophages.

Three 5 to 8-week-old conventional Belgian Landrace pigs originating from a PRRSV-negative, PCV2-positive farm were used. Using tissues of euthanized animals is in agreement with the statement of the Local Ethical and Animal Welfare Committee of the Faculty of Veterinary Medicine of Ghent University. Collection and culture of the nasal mucosa explants were performed as described before [22]. Nasal mucosa was stripped from the nasal septum and conchae and then immediately placed in transport medium containing phosphate buffer saline (PBS), 0.1 mg/mL gentamicin (Invitrogen, Gent, Belgium), 0.1 mg/mL streptomycin (Certa, Eigenbrakel, Belgium), and 100 U/mL penicillin (Continental Pharma, Puurs, Belgium). Afterwards, the tissues were cut in small square pieces (10 mm^2^), placed in six-well plates with the epithelial side facing up on a fine-meshed gauze and cultured for 24 h (37 °C, 5% CO_2_) at an air-liquid interface with serum-free medium containing 50% DMEM (Invitrogen), 50% Ham’s F-12 GlutaMAX (Invitrogen) and supplemented with 0.1 mg/mL gentamicin (Invitrogen), 0.1 mg/mL streptomycin (Certa), 100 U/mL penicillin (Continental Pharma) and 0.01 mg/mL fungizone (Bristol-Myers Squibb, USA). In order to inoculate only at the epithelial side, a polarized culture model was set up as illustrated in Figure 1, using a technology described by others [26]. PAM were seeded in a six-well plate and after 24 h of culture (37 °C, 5% CO_2_) the cells were overlaid with 3 mL of an agarose solution consisting of a 1:1 mix of 50% of sterile 6% agarose (low gelling agarose, Sigma, Diegem, Belgium) and 50% of 2× Ham’s F-12 GlutaMAX medium supplemented with 0.2 mg/mL streptomycin (Certa), 200 U/mL penicillin (Continental Pharma) and 0.2 mg/mL gentamicin (Invitrogen). Subsequently, the tissue explants were transferred from the gauzes on the solidified agarose solution and the exposed lateral edges were sealed with additional 4% agarose solution. Finally, the explants were incubated in the presence of 500 μL of culture medium at 37 °C, 5% CO_2_.

PAM were collected by lung lavage [5] and seeded (1 × 10^6^/mL per well) into 24-well plates for 24 h in RPMI-1640 supplemented with 10% fetal calf serum (FCS), 2 mM L-glutamine, 0.1 mM non-essential amino acids, 1 mM sodium pyruvate and a mixture of antibiotics. Monocytes were isolated from porcine peripheral blood mononuclear cells (PBMC) by Ficoll-paque gradient centrifugation as described by the manufacturer (Pharmacia Biotech). Afterwards, cells were seeded into 24-well plates in the same medium that was used for PAM for 2 h at 37 °C in a 5% CO_2_ atmosphere. Non-adherent cells were gently removed and the adherent monocytes were cultured in the same conditions for 24 h. Finally, for the generation of moDC an existing method was adapted [15]. Monocytes were cultured at 37 °C and 5%CO_2_ for 4 days in the presence of 20 U/mL recombinant porcine granulocyte-macrophage colony-stimulating factor (GM-CSF) and 100 U/mL IL-4. Viability and morphology of cells were monitored each day under a light microscope (Leica, Germany) and all experiments were performed in triplicate.

### Evaluation of tissue viability and morphometry in polarized explants

Cilia beating and explant morphology were observed every day under a light microscope. To evaluate the viability of the polarized explants, an In Situ Cell Death Detection Kit was used (Roche Diagnostics, Switzerland) based on terminal deoxynucleotidyl transferase dUTP nick end-labeling (TUNEL). The effect of in vitro culture on the epithelial morphology and the basement membrane thickness and continuity was evaluated by immunofluorescence staining using mouse monoclonal antibodies against cytokeratin (1:50, IgG_1,_ DAKO) and collagen VII (1:50, IgG_1_, Sigma). Samples were collected at 0, 24, 48 and 72 h for analysis of viability and morphology using a confocal fluorescence microscope (Leica TCS SP2, Germany). TUNEL-positive cells were counted within the epithelium and the lamina propria in 3 sections per sample and 5 fields of 100 cells per section. Epithelial and basement membrane thickness was measured at various time points after random selection of five fields per sample.

### Viruses

A 13th passage of PRRSV European subtype 1 Lelystad virus (LV), kindly provided by G. Wensvoort (Institute of Animal Science and Health, The Netherlands), and a 3rd passage of PRRSV European subtype 3 (Lena) were used in these studies. All viral stocks were propagated in PAM and the culture medium was stored at −70 °C. Cell-free viral strains were used after centrifugation at 13 000 rpm for 10 min, filtration through a 0.45 μm filter and dilution in serum-free medium to a final titer of 10^5.5^ tissue culture infectious dose 50% endpoint (TCID_50_)/mL [29].

### Virus inoculation and sample collection of nasal explants and monocytic cells

Explants were inoculated with 500 μL of PRRSV strains at a titer of 10^5.5^ TCID_50_/mL for 1 h at 37 °C in the presence of 5% CO_2_. Afterwards, they were washed three times with medium and further incubated with 500 μL of medium. Samples were collected at 0, 24, 48 and 72 hpi and medium was collected for virus titration. The explants were cut in two equal parts. The first part was used for immunofluorescence analysis and the second part for virus titration. To exclude virus transmission through the agarose layer, alveolar macrophages from the bottom of each well were subjected to PRRSV-specific immunoperoxidase staining [5]. No PRRSV-positive cells were found at any time-point.

PAM, monocytes and moDC were inoculated with PRRSV at a multiplicity of infection (MOI) of 0.1 virions/cell. After 1 h of incubation at 37 °C and 5% CO_2_, the inoculum was removed and the cells were washed three times with RPMI-1640 before further culture. Cells and medium were collected at different time points for viral antigen quantification and virus titration.

Culture medium, tissue suspensions and monocytic cells were titrated on PAM in quadruplicate and a final TCID_50_ was determined after subjecting the cells to a PRRSV-specific immunoperoxidase staining to analyse the presence of PRRSV positive cells.

### Immunofluorescence microscopy

To quantify PRRSV-positive cells in the nasal mucosa, several 9 μm cryosections were made at a distance of 30 μm between each other and fixed in 100% methanol at −20 °C for 15 min. Samples were subsequently incubated with the PRRSV N-specific monoclonal antibody 13E2 (1:25, IgG_2a_) and a secondary goat anti-mouse IgG_2a_ antibody (1:500, Invitrogen) [18]. To reduce the background signal, 10% negative goat serum was included for blocking during each step. Cell nuclei were stained with Hoechst (10 μg/mL, Hoechst 33342, Invitrogen) for 10 min at RT. A mouse monoclonal antibody against gB of PRV (1C11, IgG_2a_) [30] was used as negative control at the same dilution as the monoclonal antibody 13E2. Analysis was performed in a minimum of 25 sections with 5 fields per section. PRRSV-positive cells were counted within regions of interest (ROIs) including the epithelium and the lamina propria.

To identify, localize and quantify the different cell types that were infected by PRRSV, double or triple immunofluorescence (IF) stainings were performed. Mouse monoclonal antibodies were used against CD163 (2A10/11, 1:500, IgG_1_, AbD Serotec, Dusseldorf, Germany) and porcine sialoadhesin (41D3, 1:2, IgG_1_ and 262B, 1:2, IgG_2b_) [31]. To detect viral proteins, a biotinylated form of the N-protein specific monoclonal antibody 13E2 was used. Isotype-specific secondary antibodies and streptavidin conjugated with FITC, Alexa Fluor 594 or Alexa Fluor 350 (Invitrogen) were used to reveal the different markers. Cell nuclei were stained with Hoechst and to confirm the specificity of each antibody, negative isotype-specific control antibodies were used: 13D12 against gD of PRV (IgG_1_) [30], 1C11 against gB of PrV (IgG_2a_) and F190 against the capsid protein of PCV2 (IgG_2b_) [32]. Countings were made in a minimum of 3 sections and three microscopic fields (300×) per section for each marker with the sections being located 100 μm from each other.

PAM, monocytes and moDC were fixed in ice-cold methanol at −20 °C for 15 min and PRRSV-positive cells were visualized using the same technique as for the cryosections. The percentage of PRRSV-positive cells was determined for the different monocytic cell types. For each condition two independent researchers analyzed a total of 600 cells at a magnification of 400×. Analysis for all the samples was performed using a Leica TCS SP2 laser-scanning confocal microscope (Leica Microsystems GmbH, Wetzlar, Germany).

### Statistical analysis

Data were analyzed with GraphPad Prism5 software (GraphPad Software Inc., San Diego, CA, USA). Analyzed data for statistical significance were first subjected to a Shapiro-Wilk test and then a multiparameter t-test was performed. All results shown represent means and standard deviation (SD) of three independent experiments. Results with *P*-values ≤ 0.05 were considered statistically significant.

## Results

### Evaluation of tissue viability and morphometry in polarized explants

**Figure 2:**
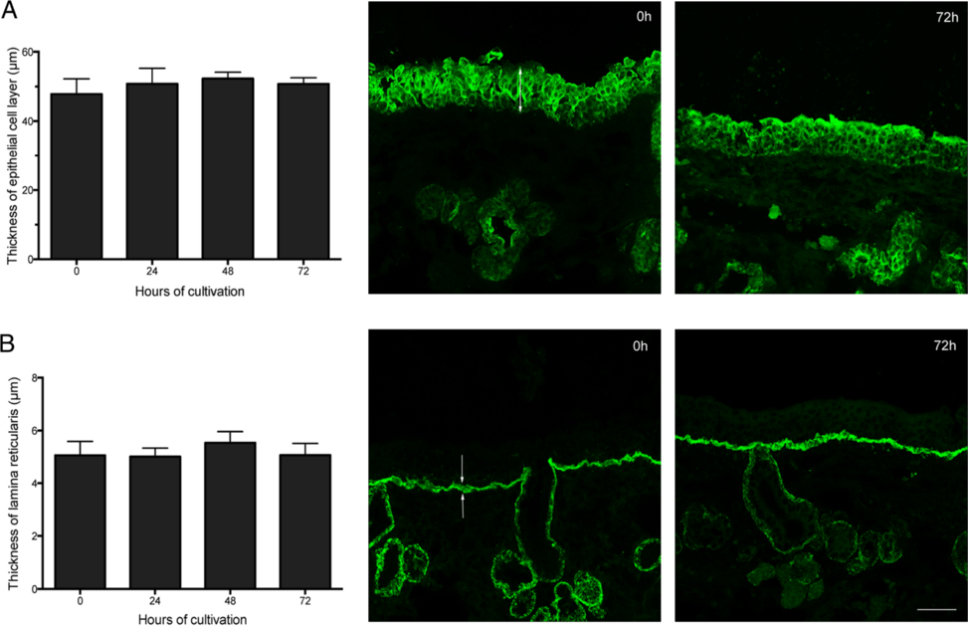
**Evaluation of the thickness of the epithelium and lamina reticularis by immunofluorescence stainings.** Average and representative confocal microscopic images of epithelium (indicated by arrow) **(A)** and lamina reticularis (indicated by arrows) **(B)** are presented. Data are represented as means ± SD of three independent experiments. Scale bar = 50 μm.

To evaluate the effect of in vitro cultivation on the viability of nasal mucosa explants, the percentage of TUNEL-positive cells was calculated at 0, 24, 48 and 72 h after incubation (Table 1). Differences in number of apoptotic cells were not observed within the epithelium and only a small increase, not statistically significant, was observed in the lamina propria at 72 h of cultivation. The thickness of the epithelium and the lamina reticularis did not change during the time of cultivation. Representative images and evaluation of morphology are illustrated in Figure 2.

**Table 1:** Evaluation of the effect of in vitro culture on the viability of nasal mucosa explants by TUNEL staining at different hours of cultivation

**Percentage (%) of TUNEL-positive cells at…h of cultivation**				
	**0 h**	**24 h**	**48 h**	**72 h**
Epithelial cells	0.2 ± 0.1	0.5 ± 0.1	0.9 ± 0.6	0.6 ± 0.3
Lamina propria	2.1 ± 0.6	3.4 ± 0.5	2.0 ± 0.4	5.3 ± 0.9

Data are represented as means ± SD.

### Virus production and quantification of PRRSV-positive cells in nasal mucosa explants

**Figure 3:**
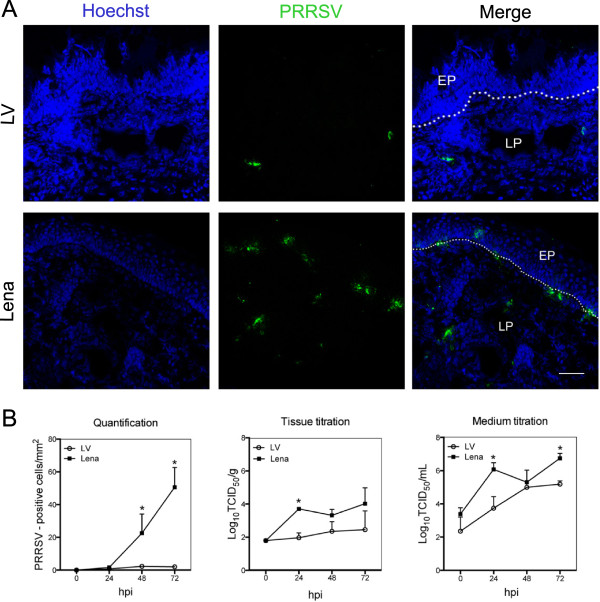
**PRRSV replication characteristics in porcine nasal mucosa explants at different hours post inoculation. (A)** Fluorescence microscopical images of nasal mucosa tissues inoculated with PRRSV strains LV and Lena at 72 hpi. PRRSV inoculated explants were immunostained with mouse anti-nucleocapsid and goat anti-mouse IgG_2a_ (green). Nuclei are visualized with Hoechst staining (blue). EP: epithelium, LP: lamina propria. White lines indicate the border between the lamina propria and the mucosal epithelium. Scale bar = 50 μm. **(B)** PRRSV-positive cells were counted in 25 sections. Tissues and medium were collected to study viral production. Error bars show ± SD and a Student’s *t*-test was performed to evaluate significant differences between samples, *denotes a *P* value ≤ 0.05.

The presence of PRRSV infected cells was determined by indirect immunofluorescence of cryosections from nasal explants collected at 0, 24, 48 and 72 hpi (Figure 3). In order to study the pattern of infection, regions of interest (ROIs) were set including the epithelium and lamina propria. At 24 hpi, single Lena-positive cells (1 to 2 cells/mm^2^) appeared in the lamina propria while no LV-positive cells were detected. At 48 hpi, the number of Lena-positive cells gradually increased with cells being located in the epithelium and in the lamina propria. Besides single positive cells, clusters of 2 to 5 cells were also observed in these areas. Only a few single positive cells (1 or 2 cells per mm^2^) were detected in LV-infected tissue, all of them located in the lamina propria. The largest number of Lena-positive cells was observed at 72 hpi (52 cells/mm^2^), while the number of LV-positive cells remained low (2 to 4 cells/mm^2^). Immunostainings of Lena-infected explants showed that expanded large clusters had accumulated within or close to the epithelium and lamina propria.

Virus titration of the 20% tissue homogenates revealed a 100-fold higher virus production rate for Lena (10^4^ TCID_50_/g) compared to LV (10^2^ TCID_50_/g) at 72 hpi. The virus titer remained stable for LV while it increased in Lena-infected explants over time (Figure 3b). In addition, virus titration of the medium, showed for Lena a 100-fold higher replication titer at 24 hpi. At 48 hpi, both strains reached the same titer of 10^5^ TCID_50_/mL and at 72 hpi, viral titers for Lena and LV were 10^6.5^ TCID_50_/mL and 10^5^ TCID_50_/mL, respectively.

### Identification of PRRSV-susceptible cells in nasal mucosae

**Figure 4:**
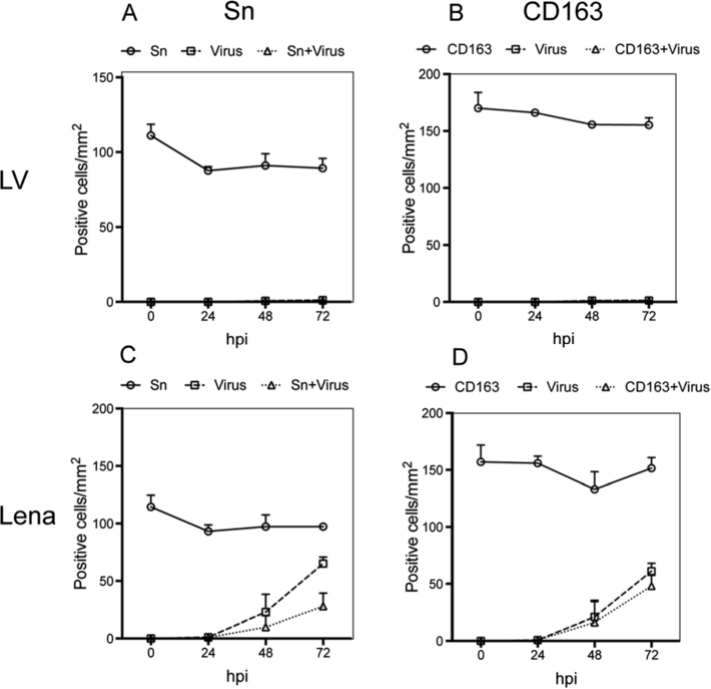
**Identification of PRRSV LV and Lena infected cells.** Nasal mucosa explants were inoculated with PRRSV strains and cultivated for 0, 24, 48 and 72 h. Cryosections were made, fixed, and co-immunostained with antibodies against PRRSV N-protein and Sn **(A**, **C)** or CD163 **(B**, **D)**. Cells were quantified within ROIs including the epithelium and lamina propria. Data are represented as means ± SD.

**Figure 5:**
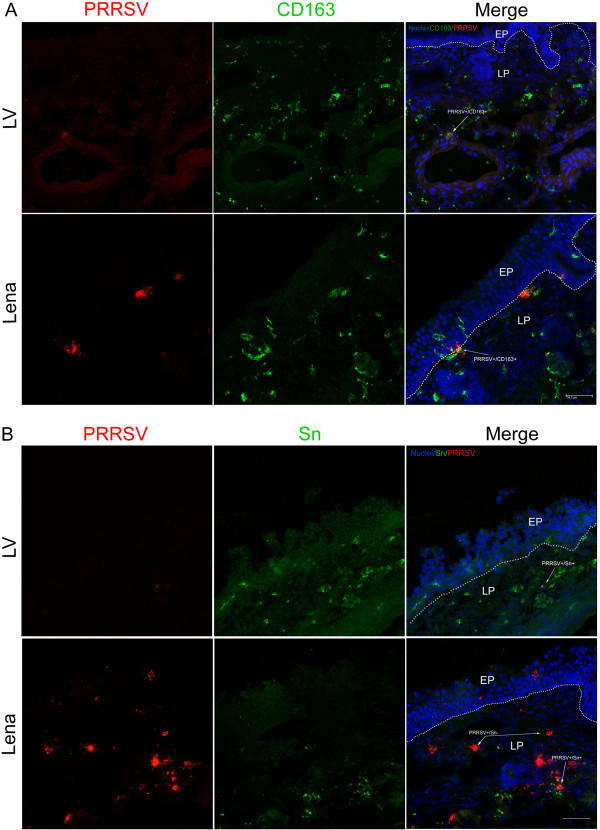
**Double immunofluorescence stainings of nasal mucosa explants.** Tissue samples were sectioned (9 μm) and co-immunostained for PRRSV N-protein (red) and markers for **(A)** CD163 or **(B)** Sn (green) at 72 hpi. EP: epithelium, LP: lamina propria. White lines indicate the border between the lamina propria and the mucosal epithelium. White arrows show CD163^+^PRRSV^+^, Sn^+^PRRSV^+^ or Sn^-^PRRSV^+^ cells. Scale bar = 50 μm.

Double IF stainings were performed for identification of PRRSV-susceptible cells. The number of cells expressing CD163 remained stable for both LV and Lena infected explants at around 150 cells/mm^2^ (Figure 4b, d). In LV-inoculated explants, all the detected LV-positive cells during cultivation were CD163^+^ and were located at the lamina propria area (Figure 4b). In Lena-inoculated tissues, viral antigen positive cells were first detected at 24 hpi and all of them were positive for CD163 (Figure 4d). At 48 hpi, 79% of the Lena-positive cells were CD163^+^. The Lena-positive cells that were CD163^-^ were located within or close to the epithelium and this distribution remained the same at 72 hpi (Figure 5a). The number of positive cells increased at 72 hpi but the percentage of viral antigen positive cells that were CD163^+^ remained stable at 76%. Clusters of infected cells in Lena-infected tissues consisted of central CD163^+^ cells and CD163^-^ cells at the periphery. Within the epithelium, single CD163^+^ cells with long dendrites were observed, resembling DC. The 75% of Lena-positive cells located in the lamina propria were CD163^+^ and were found in the proximity of secretory glands and veins.

The number of Sn expressing cells was stable for both strains during cultivation (approximately 95 cells/mm^2^) and no *de novo* upregulation was observed (Figure 4a, c). The Sn^+^ cells were located in the lamina propria and only a small number of Sn^+^ cells (4.8/mm^2^) were detected within or close to the epithelium (Figure 5b). In all LV-infected explants tested, 99% of the antigen positive cells were found positive for Sn (Figure 4a). At 48 hpi, the number of Lena-positive cells was 23 cells/mm^2^ and only 43% of the cells were Sn^+^ (Figure 4c). All the Sn^-^ cells were located close to or within the epithelium (Figure 5b). At 72 hpi, the number of Lena-positive cells raised slightly but the percentage of positive cells that were Sn^+^ remained constant at 43%.

Triple IF stainings were performed with antibodies against CD163, Sn and PRRSV N-protein. In Lena-infected tissues, both CD163^+^Sn^-^PRRSV^+^ and CD163^-^Sn^-^PRRSV^+^ cells were observed within or close to the epithelium, whereas CD163^+^Sn^+^PRRSV^+^ cells were found only in the lamina propria. At 48 hpi, the number of CD163^+^Sn^-^PRRSV^+^ cells was 7/field and the number of CD163^+^Sn^+^PRRSV^+^ cells was 4/field. At 72 hpi this proportion changed and the number of CD163^+^Sn^+^PRRSV^+^ cells was 8/field and the number of CD163^+^Sn^-^PRRSV^+^ cells was 4/field. Finally, CD163^-^Sn^-^PRRSV^+^ cells were detected at the periphery of clusters at 48 hpi. The number of these cells was 1.6/field and increased to 2.6/field at 72 hpi. In LV-infected explants, all infected cells had a phenotype of CD163^+^Sn^+^. At 72 hpi, the number of CD163^+^Sn^+^PRRSV^+^ was 2/field. All of these cells were located in the lamina propria.

### PRRSV infection kinetics in PAM, monocytes and moDC

**Figure 6:**
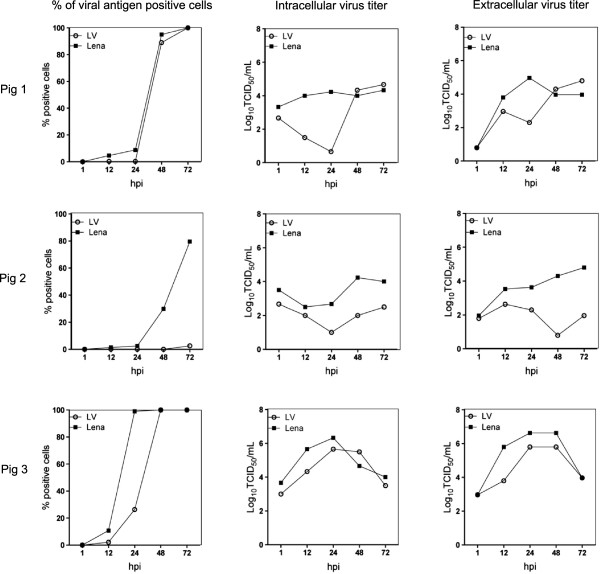
**Quantification of PRRSV-positive cells and virus titers in pulmonary alveolar macrophages (PAM).** Quantification of viral antigen positive cells is expressed as percentage (%). Intracellular and extracellular titers are expressed in a log_10_ scale. Experiments were performed with cells from three different pigs.

Infection kinetics of LV and Lena in PAM showed a different pattern in the three pigs (Figure 6). For pig 1, both strains followed the same replication kinetics reaching infection in 100% of cells after 72 hpi. Lena showed a 100-fold higher virus titer than LV at 24 hpi but at 72 hpi both strains reached a titer of 10^4^ TCID_50_/mL. For pig 2, LV-positive cells were found only at 72 hpi (5%). Lena-positive cells were already found at 24 hpi (6%) and increased at 72 hpi (80%). LV virus titers remained low (10^1.5^-10^2^ TCID_50_/mL) while Lena virus titers were over 10^4^ TCID_50_/mL. Finally in pig 3, Lena already infected 100% of the PAM at 24 hpi whereas LV reached that level only at 48 hpi. Lena showed a 2 to 10-fold higher viral titers at 24 hpi compared to LV. Both strains reached the same titer of 10^4^ TCID_50_/mL after 72 hpi.

**Figure 7:**
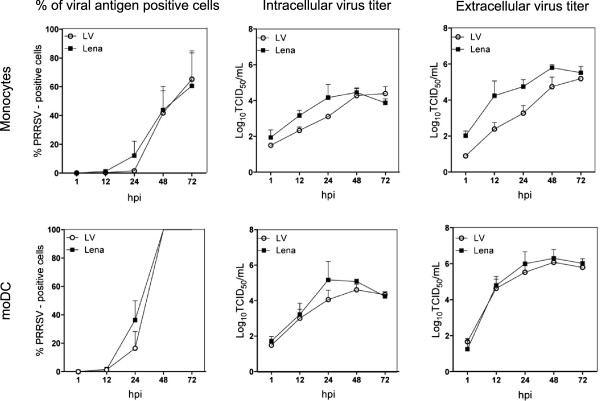
**Quantification of PRRSV-positive cells and virus titers, in cultured monocytes and monocyte-derived dendritic cells (moDC).** Cells were inoculated with PRRSV strains LV and Lena at a MOI of 0.1 for 1 h. Experiments were performed with cells from three different pigs and data are represented as means ± SD.

In cultured monocytes and moDC, replication kinetics were similar for both LV and Lena. At 72 hpi, the average percentage of infection was 60% in monocytes and 100% in moDC for both strains. Virus production in monocytes showed for Lena a 10-fold higher titer than LV at 48 hpi but at 72 hpi, both strains showed similar titers. Titrations in moDC showed no differences for LV and Lena (Figure 7).

## Discussion

In the present study it was shown that European PRRSV strains subtype 1 (LV) and subtype 3 (Lena) differ in their capacity to enter the body at the nasal mucosa: (1) the epithelial cell barrier of the nasal mucosa seems to be rather resistant to LV infection as only a few infected cells were observed at 72 hpi, all of them located in the lamina propria; (2) Lena was able to replicate much more efficiently than LV in the nasal mucosa and formed clusters of cells in the epithelial cell layer and the lamina propria; (3) LV was able to infect only CD163^+^Sn^+^ cells whereas Lena showed a wider cell tropism as it also replicated in CD163^+^Sn^-^ and to a lesser extent in CD163^-^Sn^-^ cells; (4) an alternative co-receptor might be involved in the spreading of this highly virulent PRRSV strain to other monocytic subtypes.

Studies with similar explant systems have been performed with different tissues of the respiratory tract in different animal species to investigate virus transmission and cell tropism [22-26]. To examine the behavior of PRRSV in nasal mucosa, a polarized nasal mucosa explant system was generated. Agarose was used to cover the bottom and the lateral edges of the explant, having only the apical surface free for inoculation. The epithelial thickness and the basement membrane integrity were preserved after three days of culture, in serum-free conditions. In this way, the natural route of infection was mimicked. This polarized nasal mucosa explant system allowed us to study the local PRRSV transmission and primary events during the early stages of PRRSV infection.

At 72 hpi, Lena was found to infect twenty times more cells than LV in the nasal mucosa (Lena: 51 positive cells/mm^2^, LV: 2.5 cells/mm^2^). In LV-infected tissues, mainly single infected cells were detected in the lamina propria, whereas in Lena-infected explants cell foci were also observed after 48 hpi. The small number of LV-infected cells in our experiments was in agreement with previous work, where no viral antigen positive cells were detected in the nasal mucosa after intranasal inoculation of specific-pathogen-free pigs with LV [33]. In the present study, virus titrations showed a 100-fold higher titer for Lena at the end of the experiment (72 hpi). The higher replication level that was observed with Lena correlates with previous in vivo studies where this strain was found to be more virulent than LV. Lena was secreted at high virus titers in nasal swabs (mean peak titer: 10^5.6^ TCID_50_/100 mg) and 100-fold higher titers were detected in serum (mean peak titer: 10^6.1^ TCID_50_/mL) compared to a recent European subtype 1 strain (mean peak titer: 10^4^ TCID_50_/mL) [8]. This higher virus replication coincided with severe clinical signs such as anorexia, listlessness and high fever lasting two to three weeks [8]. In another in vivo study, higher viral titers seven days after inoculation were also found for Lena in serum (10^5.4^ TCID_50_/mL) compared to LV (10^3.8^ TCID_50_/mL) and fever was observed only in Lena infected pigs [34].

In general, PRRSV shows a narrow cell tropism for cells of the monocytic lineage. Infection is primarily found in specific subtypes of macrophages located in lungs, lymph nodes, tonsils, spleen, liver, Peyer’s patches, thymus, myometrium, endometrium and placenta [9,35-39]. CD163 and Sn are two identified entry mediators for PRRSV in macrophages [19]. Sialoadhesin is a macrophage-restricted siglec that has been identified as PRRSV receptor responsible for binding and internalization [16]. CD163 is a type 1 transmembrane glycoprotein present in specific subtypes of monocytes, macrophages and DC [40]. It plays a role in viral uncoating and genome release [17]. It is already known that monocytic cells lying within the mucosal surfaces form a network in order to capture antigens, communicate with each other and process the antigens to T-cells [41]. Immunofluorescence microscopy in non-infected nasal mucosa explants revealed that CD163^+^ cells are in close proximity to each other and equally distributed within the epithelium, the lamina propria and the submucosa. Cells that are located within the epithelium showed extended dendrites displaying dendritic cell morphology. Conversely, Sn^+^ cells, were found to be equally distributed in the lamina propria and the submucosa. Only a small number of cells with this phenotype was present in the epithelial cell layer (5.2 cells/mm^2^). Cell identification experiments revealed a different cell tropism for LV and Lena. Our data suggest that the nasal mucosa is not one of the main entry portals for LV. It was shown that this strain has a restricted cell tropism for CD163^+^Sn^+^ macrophages, which are present in the lamina propria. It is thought that LV is sporadically taken up by a CD163^+^Sn^+^ macrophage and afterwards migrates to the lamina propria of the mucosa. On the contrary, Lena showed a wider cell tropism and productively infected CD163^+^Sn^-^ cells that form a dense network within the epithelium and under the basement membrane, and used them as a “trojan horse” in order to get free access to the deeper parts of the mucosa.

The single layer of columnar epithelial cells makes it easier for the nasal mucosa antigen presenting cells (APC) to extend protrusions into the airway lumen, capture antigens and migrate, compared to oral and vaginal mucosal surfaces that consist of five to seven cell layers [21,24]. After the antigen uptake, the APC regulate the degradation of the basement membrane in order to facilitate the dissemination of the pathogen to the blood circulation and other organs [20]. Unfortunately, none of the previous studies in human or other nasal mucosa tissues has included CD163 and Sn as a phenotype marker [42-44]. We believe that the CD163^+^Sn^-^ cells that are present in the porcine nasal mucosa have a specific function in antigen capturing and presentation. Additional studies are needed to further characterize these different cell types and test their properties in functional assays.

In this work, it was shown that LV-positive cells, were single infected cells located in the lamina propria and that 97% of them were CD163^+^Sn^+^. Remarkably, in Lena infected tissues large clusters of cells were formed after 48 hpi. More than 50% of these cells were CD163^+^Sn^-^ and that was the phenotype of the majority of the cells in clusters within or close to the epithelium. CD163^-^Sn^-^ cells were also found at the periphery of the clusters indicating that Lena is capable of spreading to bystander cells. It was shown that PRRSV induces apoptosis at the early stages of infection and that in vivo apoptotic cells, are engulfed by phagocytes [45,46]. Therefore, it is possible that bystander cells are infected by the uptake of apoptotic bodies that contain infectious viral particles. However, viral spreading to other cell types through phagocytosis of apoptotic remains should occur with LV as well. In the results, it was shown that infection seems to start from CD163^+^Sn^+^ cells and in Lena strain it spreads later to other cell types. This fact could be another indication that Lena uses an alternative receptor for viral entry or up-regulates specific adhesion molecules, which promote extensive viral spreading.

The dense network of monocytic cells is ideal to protect the host against invasion of pathogens into the lamina propria. The effective replication of PRRSV strain Lena may alter the function of the immune cells in the local microenvironment and change the mucosal homeostasis. A big drawback of the clustering of CD163^+^ cells is that the migrating monocytic cells may leave “open doors” for other upper respiratory pathogens to invade through the mucosa, establish a secondary infection and enhance the severity of the disease [47,48]. Previous studies have shown that PRRSV (Lena)-infected pigs allow bacteria to enter, causing sepsis and leading to death [8].

In order to compare nasal mucosa monocytic cells with primary target cells from the lungs and the blood compartment, PAM, cultured circulating monocytes and moDC were enclosed. No changes were observed in replication rates between LV and Lena in isolated monocytes from PBMC and in moDC. The replication of PRRSV strains to these cell types correlates with previous studies, but since there are functional and phenotypic differences between subpopulations of monocytic cells in different tissues, further studies with primary cells need to be done to evaluate better these results [11-15]. In PAM, the susceptibility of the two strains seemed to be pig-dependent and three patterns of infection were observed. In pig one both strains showed a similar pattern of infection whereas in pig two, LV did not replicate while Lena could replicate efficiently. Finally, in pig three Lena was able to reach higher levels of replication at early timepoints (12 and at 24 hpi). A possible explanation for the different patterns of replication observed in PAM could be the genetic background of the viral isolate and the host, which was shown before to affect the outcome of PRRSV infection [49]. Furthermore, the pigs that were used in this study were seropositive for PCV2. Therefore, a possible M1/M2 macrophage polarization that had affected the lung environment it cannot be excluded, but even then, Lena was able to replicate to all the three pigs efficiently. To the end, in this study a 13th passage of LV and a 3rd passage of Lena was used. All viral stocks were grown on PAM, which is the primary cell target of the virus. Since the virus is naturally designed to infect this cell type, it is unlikely that the passage history would have influenced the receptor preference and the outcome of the results.

**Figure 8:**
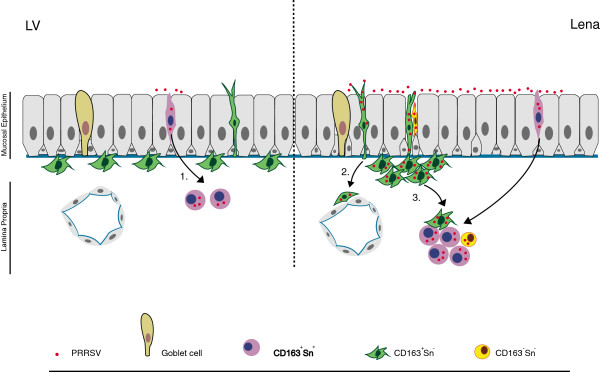
**Model of PRRSV invasion and spread in porcine nasal mucosa.** The porcine nasal mucosa consists of a ciliated pseudostratified columnar epithelium, an underlying basement membrane and the lamina propria [22]. Different mechanisms of invasion were observed between PRRSV strains LV and Lena. LV mainly infects CD163^+^Sn^+^ cells. The small number of Sn^+^ cells (5.2 cells/mm^2^) that are present within the epithelial cell layer does not allow LV to reach high levels of replication. Only 2.5 LV infected cells/mm^2^ were observed at 72 hpi, all of them located in the lamina propria. 97% of the LV-infected cells had a phenotype of CD163^+^Sn^+^ (1.). Lena showed a wider cell tropism. This strain infects both CD163^+^Sn^+^ and CD163^+^Sn^-^ cells in order to migrate into the depth (2.). Lena was found to spread easily from one cell to another, forming after 48 hpi, clusters of mainly CD163^+^Sn^-^ infected cells within the epithelial cell layer and CD163^+^Sn^+^ in the lamina propria. These clusters expanded at 72 hpi and CD163^-^Sn^-^ cells were detected at the periphery of them (3.).

In summary, different mechanisms of invasion were observed between the two PRRSV strains in nasal mucosa (Figure 8). On the one hand, LV susceptibility was mainly restricted to CD163^+^Sn^+^ cells, which were located in the lamina propria. The small number of Sn^+^ cells (5.2/mm^2^) that are present within the epithelial cell layer does not allow LV to reach high levels of infection. Only 2.5 LV-infected cells/mm^2^ were observed at 72 hpi, all of them located in the lamina propria and the 97% of them had a CD163^+^Sn^+^ phenotype. On the other hand, Lena was found to hijack CD163^+^Sn^+^ and CD163^+^Sn^-^ cells lying within or close to the epithelium in order to gain access to deeper parts of the mucosa. After 48 hpi, clusters of mainly CD163^+^Sn^-^ Lena-infected cells were observed within or close to the epithelium and clusters of CD163^+^Sn^+^ cells were observed in the lamina propria. These clusters expanded at 72 hpi, and to a lesser extent than the other phenotypes, CD163^-^Sn^-^ cells were detected at the periphery of them.

Finally, we conclude that Lena is a highly virulent PRRSV strain that can replicate more efficiently and faster than LV, in nasal mucosa and alveolar macrophages respectively. The ability of Lena to replicate in Sn negative cells suggests the use of an additional receptor for this strain. No significant differences between LV and Lena were observed in PBMC isolated monocytes and in moDC.

## Competing interests

The authors declare that they have no competing interests.

## Authors’ contributions

ISF carried out the optimization of the organ culture, performed the experiments, analyzed the data and wrote the manuscript. MV and JC performed the experiments with PAM, monocytes and moDC. HJN conceived and designed the study, coordinated the work and helped in writing the manuscript. All authors read and approved the final manuscript.
